# Autonomic cardiovascular mechanisms linked to stress in dental practice

**DOI:** 10.1038/s41415-025-9459-8

**Published:** 2026-03-27

**Authors:** Milena De Felice, Vitor de Carvalho Moreno das Neves, Camila Megale Almeida-Leite, Arshad Majid, Marco Antônio Peliky Fontes

**Affiliations:** 628729619250594310445 School of Clinical Dentistry, The University of Sheffield, UK; Neuroscience Institute, The University of Sheffield, United Kingdom; 216465852384564430501 School of Clinical Dentistry, The University of Sheffield, United Kingdom; 965326722996619233636 Department of Morphology, Institute of Biological Sciences, Federal University of Minas Gerais, Belo Horizonte, Brazil; 527895738177622878099 Sheffield Institute for Translational Neuroscience, The University of Sheffield, UK; Neuroscience Institute, The University of Sheffield, United Kingdom; 832649199300277743237 Hypertension Laboratory, INCT Nanobiofar, Department of Physiology & Biophysics, Belo Horizonte, Brazil

## Abstract

**Background** Abundant evidence from human and animal studies indicates that the sympathetic response during mental stress preferentially involves the heart. Acute or chronic stress can lead to cardiovascular disease, including hypertension and cardiac arrhythmias. Stressors in dental practice are numerous, including drill noise, sight of a needle and pain. Numerous studies indicate that many adults suffer from dental fear or dental anxiety, and many develop dental phobia. Fear, anxiety or phobia are associated with increases in heart rate, changes in systolic blood pressure, and decreased oxygen saturation. This combination of factors poses challenges for the cardiovascular system and may explain why severe cardiac perturbations are observed in some patients receiving dental treatment.

**Aim** We review the central sympathetic pathways and mechanisms involved in the cardiovascular response to mental stress in the context of dental practice.

**Methods and results** Through a comprehensive literature review we present a functional model that illustrates how dental stressors result in autonomic cardiovascular changes. The focus is on medullary and supramedullary regions mediating sympathetic output during stressful situations as well as for integrating the somatosympathetic reflex, the active reflex that controls blood pressure during painful stimulation.

**Conclusion** Concurrent activation of specific groups of sympathetic premotor neurones by both mental stress and nociceptive afferent input may underlie the abnormal cardiac sympathetic output triggered by dental stressors, potentially contributing to the initiation of cardiac arrhythmias.

## Key points

- The heart is the main target of the sympathetic nervous system in stressful situations.
- Acute or chronic stress can lead to cardiovascular alterations, including hypertension and cardiac arrhythmias.
- Stressors in dental practice are numerous, including drill noise, sight of a needle and pain.
- This review presents a functional model that illustrates how dental stressors result in sympathetic-mediated cardiovascular changes.

## Introduction: stress, anxiety and sympathetic activation

Psychological or emotional stress is broadly defined as the subjective perception of a real or potential environmental threat. This perception triggers a coordinated array of physiological responses that, from an evolutionary perspective, served to enhance fight-or-flight efficiency and thus improve survival. Stress involves both the ‘stressor' (the provoking stimulus) and the ‘stress reaction' (the ensuing physiological responses). In modern society, these stressors can differ significantly from those encountered in the past. Within the context of this review, the sound of a dental drill serves as an example of a modern stressor. The tachycardic response observed in the patient upon hearing the drill in the waiting room illustrate a characteristic physiological reaction to the perceived threat.

It is important to note that the stressor does not need to be physically present; it can also be mental and future-oriented, manifesting as anxiety. Anxiety anticipates the presence of a real stressor or stressful scenario and is often accompanied by substantial changes in vital signs, especially in the setting of dental procedures.^1^ Both real and anticipatory (anxiety-related) emotional stressors provoke physiological responses that, if chronic or exaggerated, can adversely affect the cardiovascular health and lead to conditions such as cardiac arrhythmias and hypertension.^2^

A hallmark of both stress and anxiety is increased sympathetic activation, which elevates cardiac output and peripheral resistance.^3^ A common example is seen in dental procedures, which can cause sustained increases in sympathetic activity.^4^ This heightened activity manifests as elevated blood pressure and tachycardia and, in some instances, may be accompanied by severe cardiac arrhythmias.^1^^,^^5^

Within the context of dental practice, this review highlights the primary sympathetic pathways and mechanisms that drive the cardiovascular responses to mental stress. Our aim is to raise awareness among dental clinicians about the cardiovascular challenges and potential risks associated with the emotional stress that often accompanies dento-oral procedures, risks that, apart from specific clinical exceptions, may remain unnoticed during routine dental care.

## Anticipatory stressors in dental practice

Several common emotional stressors exist within dental clinical practice. These are often perceived thorough specific sensory pathways: 1) auditory (e.g., drill noise), visual (e.g., white coat, surgical material, dental chair, light, etc.), olfactory (e.g., strong smell or resin, sodium hypochlorite and other materials), and gustatory (e.g., lidocaine, sodium hypochlorite, etc.); and 2) somatosensorial stressors; primarily painful stimuli, e.g., tooth pain, needle pain, drilling induced pain, surgical material induced pain, oral cavity manipulation, temporomandibular joint discomfort, etc.

A previous study indicated that in adults, during standard dental restoration procedures the injection of the local anaesthetic is the most stressful of all the procedures.^4^ While, a study by Peretz and Kharouba showed that, in children and adolescent teeth cleaning produced significantly more anxiety than restoration itself.^6^ Some situations may be more complex and stressful, such as the difficulty of maintaining normal breathing pattern and swallowing during dental procedures, which are known to cause marked changes in the autonomic responses.^7^^,^^8^ Therefore, many procedures must be considered as potential stressors in dental clinical practice.

Anticipatory anxiety is also equally important and can be initiated at home or in the waiting room. Recent data from Hill and colleagues showed that many adults in the UK experience dental procedures anxiety (~36% of women and ~20% of men for tooth drilling).^9^ Critically, 12% of these adults unfortunately experience extreme dental anxiety. This is of particular concern given the growing evidence that dental anxiety is associated with other psychiatric disorders,^10^ that are rapidly increasing in the urbanised areas.^11^^,^^12^

Given that all dental procedures must be considered potential stressors, it follows that the physiological responses associated with stress and anxiety are highly relevant in this context. Sympathetic activation is a hallmark of anxiety leading to increased cardiac output and peripheral resistance. For example, it is known that sustained and abnormal increases in sympathetic activity triggered by dental procedures can lead to cardiac arrhythmias even before the initiation of a dento-oral procedure.^5^^,^^13^ Notably, heightened sympathetic tone in anticipation of dental treatment has been particularly evident in vulnerable populations. For instance, highly anxious children showed significantly elevated baseline sympathetic activity in response to dental stressors.^4^ Similarly, high levels of dental anxiety have been observed among female patients, patients with infrequent dental attendance, and those anticipating invasive procedures.^6^

Learned or conditioned fear may also play a significant role in the development of the anticipatory anxiety to see the dentist, particularly when influenced by parental behaviour. Research indicates that children may acquire dental fear not from direct experience, but by observing anxious caregivers or through negative stories.^14^ This form of social learning fosters a cycle of fear even before a child undergoes a dental procedure. These findings underscore the importance of recognising psychological and psychological predisposition that may exacerbate cardiovascular responses in the dental settings.

## From normal fear to pathological anxiety: defining dental fear and dental anxiety

Despite its high prevalence and clinical relevance, dental fear remains an under-recognised and infrequently addressed problem in routine dental practice. Previous reviews aimed to differentiate the emotional status in dental patients but failed to provide a clear definition, here we aim to differentiate these in this topic. Firstly, it is important to define normal fear, apart from dental stressors. Normal fear has been defined as ‘a normal reaction to a real or imagined threat and is considered to be an integral and adaptive aspect of development with the primary function of promoting survival'. It is important to highlight that, in some conditions, normal fear can progress to pathological anxiety.^15^ Experiential, maturational and genetic factors all interact in fears. While specific fears often emerge at particular developmental stages, they tend to diminish in both prevalence and intensity with age, reflecting their typically transient nature.^16^ Fear is an emotion in terms of being caused by particular patterns of threat-related stimuli, and in turn causing particular patterns of adaptive behaviours associated to physiological changes to avoid or cope with that threat.^17^ Thus, dental fear follows the same definition with the distinction that the stressor is specifically identifiable, namely, the dental procedure itself or the broader clinical environment. Triggers may include sensory cues such as the sound of a dental drill or visual stimuli like the close proximity of a masked practitioner, all of which can evoke significant emotional and physiological responses in susceptible individuals.^18^

Following this rationale, dental anxiety can be defined as the imaginary anticipation of the dental stressor and also involves physiological and behavioural changes but more from a tonic state related to prediction and preparedness. Regarding the causes, research indicates that dental fear in children is strongly associated with inadequate dental management, causing anxiety levels to increase in both the children and parents.^19^ Finally, in extreme cases particularly traumatic experiences may lead to odontophobia, described as a ‘unique phobia with special psychosomatic components that impact the dental health of the odontophobic persons'.^20^ This condition is characterised by fear or anxiety that is disproportionate to the actual threat posed by the dental context. This often leads to avoidance behaviour, psychological distress, and impaired daily functioning.^21^ Patients with severe anxiety often cannot regulate their fear, and their emotional reactions such as vomiting, crying, screaming, may interfere with the delivery of dental care, particularly during surgical procedures.^22^ Pathological anxiety is conceptualised as an exaggerated fear state, characterised by hyperexcitability of fear circuits – including, among other regions, the amygdala, insular cortex, the hypothalamus and the midbrain periaqueductal gray (PAG) and is expressed as hypervigilance, increased behavioural responsiveness to fearful stimuli, and sympathetic overactivity affecting target systems such as the cardiovascular system.^2^^,^^15^^,^^23^ It is important to note that dental anxiety or dental phobia are diagnosed using specific psychological evaluation questionaries (dental anxiety scale) and some physiological parameters such as galvanic skin reaction or heart rate.^13^^,^^24^^,^^25^^,^^26^

## Stress and cardiovascular risk

Sympathetic nerve activity comprises bursts of action potentials originated by neurones located in specific nuclei in the central nervous system. These potential firings travel through sympathetic nerves with the objective of modifying the functional activity of a target organ.^27^ Thus, organs and systems are influenced by the so called ‘sympathetic tone' or ‘sympathetic activity', that may increase or decrease depending on the physiological demand. For example, during emotional stress situations the sympathetic tone to the heart, kidney and adrenal medulla increases to organise blood flow redistribution, mainly keeping secure energy and oxygen supply for muscles, heart and brain.

Thus, the sympathetic activity, generated in brain nuclei, is driven through sympathetic nerves. The burst pattern of the sympathetic activity will reflect in the amount of noradrenaline released in a specific target organ, named noradrenaline spillover. Sympathetic activity also controls the release of adrenaline and noradrenaline from the adrenal medulla into the systemic circulation. There are different ways for measuring sympathetic activity in rodents as well as in humans.^28^

Consistent evidence indicates that the pattern of sympathetic response during mental stress preferentially involves the heart,^29^ specifically through increasing heart rate and stroke volume, similarly found for dental stressors. Previous studies have shown that, in anxious patients, the baseline heart rate may be significantly higher immediately before dental checkup^18^^,^^30^ and, in some cases cardiac alterations are observed.^31^ An interesting study by Lundgren and colleagues showed that dental phobic patients can present a marked tachycardic response when exposed to a simple dental and neutral video scenes^18^ (Table 1). Increasing heart rate and stroke volume leads to increases in systolic blood pressure. In anxious patients, cardiovascular alterations have been described during different typical dental procedures such as tooth extraction.^32^ However, under pathological conditions, dysregulated sympathetic activity directed towards the heart can lead to cardiac arrhythmias and, in case of severe emotional stress may contribute to the development of Takotsubo syndrome, a condition characterised by acute transient left ventricular dysfunction. Notably, Takotsubo syndrome has been reported following a minimally invasive tooth extraction in a patient with no prior history of dental phobia.^33^ Therefore, all general dentists and oral surgeons should critically consider the potential cardiovascular complication, even during routine or minimally invasive procedures, particularly in patients who may not exhibit overt signs of dental anxiety.

**Table 1:** Examples of reported cardiovascular alterations that may accompany patients suffering from dental anxiety

**Author, year**	**Condition or event as described by authors**	**Outcome, main findings or conclusions**
Brand *et al*., 1995	Anxious patients	Anticipation of a dental checkup increases heart rate
Jacobson *et al.*, 1996	40 older patients during an outpatient oral surgery visit	Arrhythmias were detected in 17 patients; significantly more arrhythmias occurred before administration of anesthesia
Lundgren *et al*., 2001	Dental phobia	Elevated baseline heart rate
Lundgren *et al*., 2001	Dental phobia	Marked tachycardic response when exposed to a simple dental and neutral video scenes
Liau *et al*., 2008	Younger patients with high anxiety	High anxiety, younger age, and traumatic dental history were correlated with greater increases in heart rate during the administration of local dental anesthesia
Shimoda and Takahashi, 2019	Patient under mental and physical stress associated to dento-oral surgery	Paroxysmal atrial flutter before the initiation of the dento-oral surgical procedure
Košir *et al*., 2021	Subjects with dental anxiety exposed to real life dental examination	Dental-related stimuli increases heart rate, heart rate variability and skin conductance level (increases in sympathetic activity)
Shimoda *et al*., 2021	Serious dental anxiety patient	Atrioventricular junctional rhythm is induced by an imbalance of autonomic activity owing to excessive psychosomatic stress
Takuma *et al*., 2023	Minimally invasive tooth extraction in a patient with no prior history of dental phobia	Atypical Takotsubo syndrome in a woman who reported no dental fear and required acute cardiac care in an outpatient setting
Özmen and Tasdemir, 2024	Anxious, adult patients	Positive correlations were observed between anxiety and preoperative pulse rate

Following this rationale, the clinician might assume that blocking β1 cardiac adrenergic receptors would prevent increases in heart rate and blood pressure during stressful situations. One could easily conclude that, by preventing the stress-associated cardiac changes and dampening anxiety,^34^ β1 adrenergic blockade leads to cardiovascular protection. Recent studies in rodents, however, showed that systemic administration of the β-blocker atenolol moderately attenuated the tachycardic effect to acute stress but had no effect to blunt the initial and large increase in mean arterial pressure.^2^^,^^35^ Thus, this experimental evidence suggests that cardiovascular protection is not guaranteed given that a large pressor response may still be present with systemic atenolol treatment when facing stressful situations.^2^^,^^35^ In agreement with this experimental evidence, a previous study by Schweizer *et al.* evaluated the cardiovascular and behavioural effects of two β-blockers in humans during a mental stress task. Authors found that, under β blockade treatment, diastolic blood pressure still increases in the presence of the stressor and the tachycardic effect is only attenuated. Interestingly, the increase in self-rated stress produced by the mental stress task was greater in the atenolol than in the placebo condition.^36^ On the other hand, a more recent report shows a reduction in anxiety in humans taking atenolol or other β blockers.^34^ The mechanism, as discussed below, may result from a reduced conscious perception by the patient themselves of the heart functional changes (chronotropic and inotropic responses) when facing stressful events. As previously reported, there is a correlation between anxiety and greater firing rate of individual sympathetic fibres.^37^ Moreover, the amplitude of sympathetic nerve activity in response to acute emotional stress is selectively augmented in individuals with chronic anxiety, a risk factor for the development of cardiovascular disease.^38^ Studies suggest that some individuals are more autonomic-reactive in anxiety situations, linking emotional responses to health risks, including cardiovascular risks. For example, white-coat hypertension, a phenomenon characterised by higher anxiety levels,^39^^,^^40^ is very common in children and adolescents.^41^^,^^42^

Taken together, these findings indicate that stress and anxiety modulate central sympathetic outflow in ways that can increase cardiovascular vulnerability. The neurogenic background for the origin of emotional stress-induced cardiovascular changes is now relatively well-understood.^2^

## Central sympathetic mechanisms and pathways activated during emotional stress

**Fig. 1:**
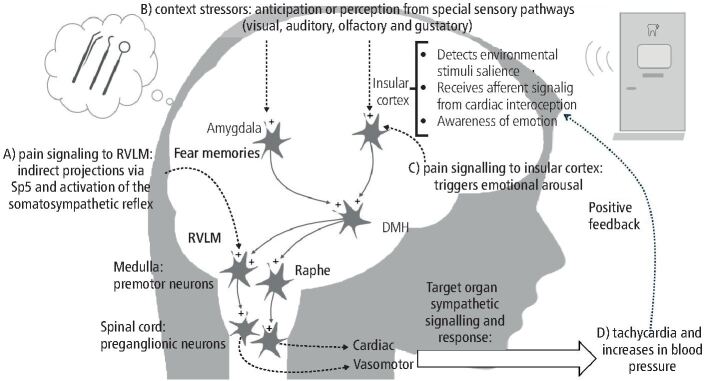
Schematic representation of the central sympathetic pathways mediating the cardiovascular alterations in response to somatic or contextual stressors (A-C). The scheme proposes a synergistic interaction between painful and contextual fear stimuli. The somatosympathetic-mediated cardiovascular responses triggered by pain originating in the mouth are integrated in the medulla likely via indirect pathways from the spinal trigeminal nucleus (Sp5). The increases in sympathetic activity to cardiovascular target organs resulting from contextual stressors involve downstream pathways that include synaptic relays in the insular cortex and amygdala; these regions are integrated, via the dorsomedial hypothalamic region, with the rostralventrolateral medulla and raphe pallidus leading to vasomotor and cardiac alterations. Conscious perception of heart rate changes (inotropic, chronotropic or even arrhythmias), via positive feedback, could magnify fearful experiences detected by the insular cortex and, in turn, enhance the cardiac sympathetic output to the heart. ‘Plus' symbols (+) indicate excitatory activity; dashed arrows represent indirect projections (see main text for details; based on previous reports).^2^^,^^47^^,^^74^^,^^94^^,^^97^ Anatomical locations are representative

Over the last two decades, studies have unravelled the central sympathetic pathways mediating the cardiovascular responses during emotional stress and these include projections from the insular cortex to downstream autonomic centres such as the dorsomedial hypothalamus and the rostral ventrolateral medulla (RVLM)^2^ (Fig. 1).

The human insular cortex is hidden from the brain's outer surface, lying in the centre of the cerebral hemisphere and forms a distinct, but entirely hidden island of cortex (hence the term ‘insular'), situated in the depth of the Sylvian fissure.^43^ There is clear evidence indicating that the insular cortex plays an important role in adjusting the sympathetic-mediated cardiovascular response associated to decision-making during emotional situations.^44^^,^^45^^,^^46^ The posterior insular cortex receives information regarding cardiac activity, working as a potential mediator of bottom-up cardiac interoceptive processing allowing interoceptive perception (anxiety or emotional stress-induced cardiac changes in this case). Interestingly, inhibition of the posterior insular cortex attenuates the anxiety-like behaviour induced by interoceptive perception of the tachycardic responses.^47^

The insula does not send direct projections to preganglionic sympathetic neurones. As we have recently demonstrated, the tachycardia mediated by insular activation is largely dependent upon a glutamatergic relay with the dorsomedial hypothalamus (DMH).^48^ The DMH is a major site relaying the cardiovascular response to stress, it is considered part of the hypothalamic defence area.^49^^,^^50^^,^^51^

On the other hand, the descending projections from the DMH are dependent on synaptic relays with the RVLM and midline raphe neurones.^2^ The DMH has also reciprocal connections with the lateral/dorsolateral midbrain PAG region,^52^^,^^53^ which is another key region involved in the integration of stress-evoked fight or flight strategies associated to cardiorespiratory responses.^54^^,^^55^

Accumulated evidence indicates that there are separate pathways for mediating the pressor and tachycardic responses evoked from the DMH and thus those triggered by emotional stress. The tachycardic response is mediated via raphe pallidus neurones located at the ventromedial medulla, and the vasomotor (vascular response) seem to be largely dependent upon RVLM neurones.^56^^,^^57^^,^^58^^,^^59^

Downstream, located in the ventral medulla, the RVLM is a key region for the maintenance of sympathetic output. The activity of vasomotor and cardiac sympathetic nerves is largely dependent on excitatory drive from sympathetic premotor neurones located in the RVLM.^60^^,^^61^ In addition, several previous studies show that this group of medullary sympathetic premotor neurones plays a role in mediating the cardiovascular response associated to stressful conditions.^62^^,^^63^^,^^64^^,^^65^ Moreover, previous consistent evidence indicates that the RVLM is a synaptic relay in the descending pathways mediating responses evoked from the DMH. The increases in blood pressure evoked by DMH activation are abolished by RVLM inhibition, while the tachycardic response is preserved.^56^^,^^57^^,^^58^ Therefore, indirectly, these findings strongly suggest the participation of RVLM neurones in mediating the increases in blood pressure evoked by emotional stressors.^60^ It is necessary to point out, however, that some previous studies showed that RVLM is not activated in response to arousal or psychological stress.^66^^,^^67^ Different methodological strategies used in all these previous studies can explain these different findings. Undoubtedly, future investigation using more modern approaches such as optogenetics and chemogenetics^68^ is necessary to further confirm the role of RVLM in stress-mediated cardiovascular responses. Despite these different findings, evidence indicates that the same circuit involved in sympathetic control described in rodents^2^ seems to be present in humans. In this regard, previous studies revealed a strong coupling between the insula, DMH, and RVLM at rest for the generation of sympathetic outflow to the maintenance of blood pressure.^45^^,^^69^

In summary, consistent evidence indicates that the sympathetic responses evoked during emotional stress situations seem to be dependent on activation of the insular-DMH pathway that in turn relays with medullary premotor neurones for adjusting the homeostatic cardiovascular responses (Fig. 1).

## Connecting the somatic and contextual components: links for understanding the relevance and the magnitude of the sympathetic-mediated cardiovascular response evoked by stressful dental approaches

A key question arises: why is dental fear so prevalent? Painful or traumatic dental experiences are often cited as the main causes, since these can establish strong and long-lasting pain-related fear memories.^70^ Thus, a painful experience would lead to contextual fear, i.e., a type of learning that associates a specific environment with a fear-inducing stimulus. In the case of dentistry, both somatic stimuli (e.g., pain) and contextual cues (e.g., sounds, visual stimuli) can trigger sympathetic and emotional responses.

To fully understand the neurobiological mechanisms driving exaggerated cardiovascular responses in the dental setting (Fig. 1), it is important to separate the origin of stressful dental-related stimuli into two components:

1. The somatic component, largely comprising nociceptive input
2. The contextual component, which includes non-nociceptive sensory cues capable of triggering autonomic and emotional reactivity.

Both components engage central autonomic circuits exposed above, including those involving the insula, DMH, and RVLM, to generate the physiological profile commonly observed in anxious or phobic patients (Fig. 1).

## The somatic component: nociception, somatosympathetic reflex, and cardiovascular integration

Somatic afferents provide a coordinated cardiovascular reaction to changes in the physical state or internal state of the body, playing a critical role in the regulation of autonomic and endocrine system during stress.^71^ These afferents contribute to homeostasis by dynamically modulating visceral organ activity through reflexive autonomic adjustment, thereby enhancing the body's resistance to external and internal stressors. In neurophysiological terms, somatic sensory response is generally divided into four modalities:

- Proprioception
- Touch
- Thermal sense
- Nociception (pain).

Each of these modalities is transduced by primary sensory neurones located in the dorsal root ganglia, which serve as the interface between the peripheral stimuli and central integration pathways. The primary afferent receptors that mediate pain are specifically named ‘nociceptors'. Dorsal root ganglion neurones are the primary afferents or sensory receptor cells for pain mediated by spinal nerves and trigeminal ganglion neurones are the primary afferents for pain felt in the face, oral cavity and head, which is mediated by the trigeminal nerve (V).^72^^,^^73^ Nociceptors are critical not only for the conscious perception of pain but also for mediating reflexive autonomic responses. For the sympathetic adjustments associated to painful experiences, upon activation, nociceptive signals ascend centrally and are integrated within the medulla oblongata, particularly in the RVLM, a major premotor nucleus for sympathetic outflow. Thus, stimulation of pain fibres increases the discharge of the postganglionic sympathetic nerves, via synaptic relay with the RVLM premotor neurones, causing sympathoexcitation and large rise in arterial pressure,^74^ a response collectively referred to as the somatosympathetic reflex (Fig. 1A).

The somatosympathetic reflex arc, which is considered segmental in nature, can be elicited even in the absence of supramedullary control, underscoring its fundamental role in autonomic regulation.^74^ In humans, activation of somatic afferents – particularly those within the trigeminal system – can evoke large increases in sympathetic tone and arterial pressure (cardiovascular response), as demonstrated in experimental and clinical studies.^75^^,^^76^ The orofacial region is especially relevant in this context, as nociceptive inputs from the trigeminal nerve project to brainstem nuclei involved in cardiovascular control such as the nucleus tractus solitarius (NTS),^77^ to the midbrain PAG,^78^ and ultimately influence the activity of rostral ventromedial pain-modulating neurones.^79^ As well as the RVLM, neurones of the rostral ventromedial medulla are also involved in cardiovascular control.^58^^,^^80^

This raises the question of how oral nociceptive input is integrated with medullary nuclei to adjust cardiovascular responses. Specifically, primary nociceptive inputs from the trigeminal nerve project to the spinal trigeminal nucleus (Sp5), located at the lower pons to cervical cord up to the C2–C4 level. The Sp5 mediates, pain, crude touch and temperature sensations.^81^^,^^82^ From Sp5, it is likely that indirect projections to medullary nuclei mediating the cardiovascular control mediate the somatosympathetic responses triggered by mouth nociceptive afferents. In this regard, it is important to point out that the Sp5 is connected with the paratrigeminal nucleus.^83^ The paratrigeminal nucleus is an input area for trigeminal afferents, a component of the spinal trigeminal complex and an orofacial nociceptive sensory processing site.^84^^,^^85^ Functional and anatomical studies show connections from the paratrigeminal nucleus to the NTS and RVLM, and thus it may serve as a relay and integration centre mediating somatosensory reflexes, evoking cardiovascular responses triggered by pain stimuli.^86^^,^^87^^,^^88^

It is important to point out that, apart from RVLM, other brain regions involved with the autonomic cardiovascular control are also activated by nociceptive stimulation. In this regard, the insular cortex, as evidenced earlier a critical hub for interoceptive processing and sympathetic modulation, is directly influenced by nociceptive stimuli integrating trigeminal nociceptive inputs, including those associated to abnormal pain.^89^^,^^90^^,^^91^^,^^92^ The insular cortex is an important brain region controlling the sympathetic autonomic output to the heart and kidneys.^93^ Responses evoked from the insular cortex are indirectly mediated via RVLM and raphe pallidus via the dorsomedial hypothalamus.^48^^,^^94^ Thus, it is likely that somatic stimuli with projections to the insular cortex are also integrated with medullary neurones, including RVLM, to be translated in motor autonomic output that leads to tachycardia and increases in blood pressure (Fig. 1).

In the context of dental procedures, the somatosympathetic reflex is of clinical importance. Nociceptive input resulting from needle insertion, drilling, or surgical manipulation directly activates orofacial nociceptors, particularly within the trigeminal nerve. When this afferent input is superimposed on an already heightened emotional stress state, it may significantly augment central sympathetic outflow. Indeed, activation of premotor neurones by emotional stressors has been shown to exacerbate cardiovascular reactivity leading to cardiac arrythmias.^95^ This synergistic interaction with the somatic, painful, component may increase the risk of acute hypertension, arrhythmias, or even stress-induced cardiomyopathies during dental interventions.

Therefore, understanding the neurophysiological basis of the somatosympathetic reflex alongside its interaction with emotional stress pathways is crucial for anticipating autonomic instability in patients undergoing invasive dental procedures.

## The contextual component: a central command sympathetic mechanism

Complementing this somatic pathway is a contextual, centrally driven component that modulates sympathetic output through cognitive-emotional circuits. A recent study showed that in patients with dental anxiety all dental-related stimuli, not necessarily painful, provoked significant increases in heart rate including visual, auditory and a dental examination. Additionally, autonomic responses did not differ between the experimental and control groups.^96^ This supports the concept of a central command mechanism, wherein emotional and cognitive factors alone can recruit autonomic cardiovascular responses. Here, the insular cortex again plays a pivotal role, functioning as a hub for stimulus salience detection, visceral interoception, and emotion processing.^97^ Fear memories are also registered in the amygdala which is an important part of fear circuit that mediates the autonomic-cardiovascular responses associated with contextual fear and emotional stress^2^ (Fig. 1B). A previous neuroimage study performed in humans showed that the auditory cortex and amygdala are activated by sounds produced by dental instrumentation.^98^

Interoceptive processing of visceral physiological signals, such as cardiac palpitations are crucial for maintaining homeostasis and to feedback emotional states. Recent findings have identified the insular cortex as a potential mediator of bottom-up cardiac interoceptive processing.^47^^,^^97^ Interestingly, Hsueh and colleagues found that optogenetic inhibition of the insular cortex attenuated the anxiety-like behaviour that was induced by a tachycardia triggered by a non-invasive cardiac pacing.^47^ These studies support early theories indicating that the interoceptive information from the heart is necessary to feedback emotional states, in particular, the James-Lange theory;^97^^,^^99^ noticing that the heart rate changes can feedforward fearful experiences increasing the cardiac sympathetic output to the heart. In summary, current evidence indicates a clear link between interoception and intensity of emotions such as the link that mediates cardiac interoception and the insular cortex activity (Fig. 1C). This loop helps to understand why negative dental experiences can result in such large increases in sympathetic output sometimes accompanied by major cardiovascular alterations.

## Strategies to minimise the cardiovascular effects resulting from emotional stressors in dental clinical practice

To mitigate cardiovascular alterations in clinical practice, some strategies have been described to be effective. Among pharmacological management, some systematic reviews have shown that intravenous or oral conscious sedation with midazolam^100^ or oral sedation with midazolam, diazepam or herbal medicines^101^ reduce anxiety and improve patient satisfaction. However, some other reviews concluded that there is insufficient evidence to determine whether pharmacological control of dental anxiety is efficient enough.^102^^,^^103^ Moreover, pharmacological intervention can have undesirable side effects, risks and increase the cost and availability of dental treatment.^104^

Nonpharmacological strategies include many psychotherapeutic behavioural techniques that can modify the patient's experience and aid in reduction of fear and anxiety through a minimally invasive approach with minor side effects. These therapies involve muscle relaxation, acupuncture, distraction, aromatherapy, virtual reality and/or audio-visual aids.^104^^,^^105^

## Conclusions

Sympathetic activity is a hallmark of stress and anxiety situations, and the heart is the main target. Given the variety of stressors present during a dental treatment sympathetic pathways are constantly activated. Activation of somatosympathetic reflex associated to contextual fear is likely activating a positive feedback and sustained loop for sympathetic responses mainly mediated by the insular cortex via medullary sympathetic premotor neurones, including the rostroventrolateral medulla (Fig. 1). Dental clinicians must be aware of the cardiovascular challenges and risks that are associated with emotional stress resulting from dento-oral procedures.

## Data Availability

Not applicable. This is a narrative review written with educational purposes; thus, conclusions were based on data previously published. All articles used are cited in the References section and respective authors are responsible for their content.
